# Supplementary material for: Relationship between Apolipoprotein E Genotype and Lipoprotein Profile in Patients with Coronary Heart Disease
Source: Molecules. 2022 Feb 18;27(4):1377. doi: 10.3390/molecules27041377 (PMC8879216; doi:10.3390/molecules27041377)
Supplement: Supplementary file 1 [file molecules-27-01377-s001.zip › molecules-1560750-supplementary.pdf]

molecules

Supplementary Materials

Table S1: Comparison of lipoprotein particle concentrations of 360 patients grouped by APOE genotype. Parameters are described by mean ± SD.

1. (nmol/L)	2. ε2+	3. ε3	4. ε4+	5. *P*
6. **VLPN***	7. 212.58±155.44	8. 167.79±76.77	9. 176.2±67.58	10. 0.008
11. **IDPN**	12. 71.17±47.89	13. 65.77±33.44	14. 67.21±31.09	15. 0.624
16. **LDPN***	17. 995.52±379.51	18. 1141.02±372.29	19. 1253.01±479.85	20. 0.004
21. **Small LDL***	22. 436.52±246.36	23. 560.29±223.37	24. 618.87±251.13	25. <0.001
26. **Medium LDL***	27. 240.46±123.81	28. 293.62±143.3	29. 330.65±179.95	30. 0.008
31. **Large LDL**	32. 316.2±125.78	33. 280.64±113.06	34. 292.17±99.88	35. 0.131
36. **LDL size***	37. 20.44±0.32	38. 20.20±0.29	39. 20.17±0.25	40. <0.001

*$P<0.05$;

Table S2: Comparison of lipoprotein compositions concentrations of 360 patients grouped by APOE genotype. Parameters are described by median (IQR).

		ε2+	ε3	ε4+	P
apo A1	Total apo A1	128.22(118.56-138.19)	122.22(110.9875-135.5075)	119.43(107.83-133.88)	0.100
	HDL	123.3(113.595-133.445)	116.6(105.635-133.4025)	115.4(102.04-128.12)	0.054
	HDL-1	19.03(15.28-27.18)	18.045(14.3075-23.605)	16.3(13.97-20.85)	0.051
	HDL-2	12.33(9.95-15.015)	12.175(9.805-14.325)	11.18(9.08-13.51)	0.035 *
	HDL-3	20.99(18.74-23.81)	19.485(16.6325-23.0375)	18.58(15.64-21.86)	0.014 *
	HDL-4	61.65(57.625-67.84)	62.825(55.8125-70.6825)	64.58(59.07-71.33)	0.482
apo A2	Total apo A2	25.2(23.585-26.865)	24.38(21.84-27.6775)	23.81(22.02-26.89)	0.537
	HDL	26.04(24.51-27.825)	25.395(22.9-28.29)	24.74(23.01-27.91)	0.522
	HDL-1	0.97(0.56-1.895)	1.065(0.57-1.7)	0.83(0.57-1.26)	0.124
	HDL-2	1.87(1.215-2.62)	1.79(1.26-2.4125)	1.48(1.26-1.95)	0.042 *
	HDL-3	4.61(3.91-5.49)	4.235(3.6175-5.1025)	4.04(3.53-4.81)	0.057
	HDL-4	14.1(12.735-16)	15.23(13.0125-17.41)	15.62(14.13-16.95)	0.128
apo B100	Total apo B100	76.14(52.525-90.65)	77.085(62.9675-94.6675)	83.92(68.68-101.74)	0.058
	VLDL	9.98(7.535-12.645)	8.525(6.215-11.45)	8.88(6.84-11.86)	0.077
	IDL	3.54(2.18-5.105)	3.27(2.4275-4.5725)	3.56(2.48-4.71)	0.734
	LDL	52.56(37.44-68.875)	59.325(47.0825-77.7825)	63.17(54.47-80.15)	0.007 **
	LDL-1	10.42(7.55-13.68)	8.66(6.9225-10.99)	9.37(7.88-11.5)	0.022 *
	LDL-2	5.45(4.445-7.555)	5.61(3.9975-7.1575)	5.81(4.5-7.96)	0.621
	LDL-3	5.63(4.035-7.77)	6.92(5.34-9.04)	7.45(5.64-9.67)	0.019 *
	LDL-4	6.77(3.83-9.695)	8.38(5.8275-11.405)	9.1(6.38-11.82)	0.006 **
	LDL-5	8.05(4.735-11.9)	11.35(8.485-15.51)	12.69(9.51-16.78)	<0.001 **
	LDL-6	13.49(9.245-19.9)	17.415(13.315-23.9275)	21.08(14.63-25.38)	0.001 **
Cholesterol	Total CH	163.74(132.735-193.31)	163.005(136.2-192.595)	173.67(149.49-195.66)	0.338
	VLDL	72.63(50.395-101.465)	86.5(67.835-108.9625)	93.5(74.75-112.13)	0.006 **
	IDL	46.07(39.485-49.3)	43.85(38.09-50.285)	42.65(37.36-48.01)	0.181
	LDL	21.17(15.15-32.41)	18.765(12.135-26.06)	20.43(13.53-26.99)	0.113
	HDL	9.68(5.49-15.02)	8.835(6.0625-12.7725)	9.93(6.54-13.79)	0.390
	VLDL-1	6.42(4.305-10.91)	5.53(3.1025-8.8125)	6.09(3.88-10.17)	0.102
	VLDL-2	2.89(1.87-5.545)	2.84(1.5325-4.3225)	3.42(2.16-5.22)	0.118
	VLDL-3	3.98(2.615-6.865)	3.62(2.105-5.715)	4.33(2.45-5.8)	0.208
	VLDL-4	5.06(3.095-7.05)	4.18(2.8625-5.7)	4.37(3.25-5.67)	0.230
	VLDL-5	1.77(1.425-2.28)	1.48(1.1225-1.92)	1.53(1.31-1.77)	0.011 *
	LDL-1	17.79(13.475-25.4)	15.12(12.0525-19.64)	16.59(14.05-20.75)	0.028 *
	LDL-2	8.04(6.22-12.22)	8.685(5.6325-12.06)	9.53(6.27-12.82)	0.750
	LDL-3	7.68(5.625-12.23)	10.24(7.525-14.5325)	11.4(7.94-15.69)	0.013 *
	LDL-4	9.18(4.855-14.82)	12.375(8.0825-17.575)	14.32(9.66-18.42)	0.007 **
	LDL-5	10.15(6.21-15.725)	15.765(10.975-22.085)	17.07(13.95-23.79)	<0.001**
	LDL-6	15.19(11.46-23.36)	20.925(16.1775-27.9075)	25.54(17.93-29.81)	<0.001**
	HDL-1	13.22(11.215-17.005)	13.45(10.7975-16.355)	12.7(10.68-15.55)	0.451

	HDL-2	7.05(5.87-9.035)	6.645(5.53-8.4025)	6.13(5.04-7.31)	0.006 **
	HDL-3	7.84(6.64-8.84)	7.25(6.0575-8.8425)	7.28(5.69-8.33)	0.126
	HDL-4	14.85(13.02-16.365)	15.315(12.7325-17.935)	15.72(13.19-17.45)	0.401
Free Choles-terol	VLDL	10.13(7.4-13.465)	8.74(6.145-11.7025)	9.4(6.73-12.58)	0.094
	IDL	3.06(1.73-4.35)	2.545(1.7-3.75)	2.99(1.94-3.97)	0.246
	LDL	23.32(16.81-30.615)	26.36(21.48-32.8875)	28.76(23.56-33.38)	0.009 **
	HDL	10.41(9.03-12.26)	10.34(9.0375-11.8975)	9.61(8.09-11.76)	0.327
	VLDL-1	2.34(1.425-3.75)	1.83(0.8975-3.4425)	2.41(1.24-3.78)	0.089
	VLDL-2	1.65(1.205-2.715)	1.385(0.7775-2.305)	1.53(1.01-2.91)	0.091
	VLDL-3	2.05(1.395-3.18)	1.66(0.9975-2.6825)	1.96(1.11-2.97)	0.128
	VLDL-4	2.67(1.69-3.5)	1.935(1.2475-2.8)	2.05(1.31-2.63)	0.035 *
	VLDL-5	0.43(0.175-0.74)	0.27(0.0775-0.5225)	0.24(0.08-0.41)	0.008 **
	LDL-1	5.59(3.965-7.695)	4.715(3.6175-6.13)	4.95(4.18-6.35)	0.059
	LDL-2	3.11(2.2-4.045)	3.11(2.21-4.1225)	3.31(2.31-4.2)	0.794
	LDL-3	3.74(3.105-4.825)	4.325(3.405-5.1925)	4.51(3.43-5.97)	0.072
	LDL-4	3.77(2.615-4.9)	4.3(3.32-5.5525)	4.88(3.58-5.8)	0.010 *
	LDL-5	3.17(1.96-4.37)	4.44(3.345-5.8325)	4.8(3.92-6.12)	<0.001 **
	LDL-6	4.02(2.72-5.32)	5.375(4.13-6.8575)	6.1(4.44-7.2)	<0.001 **
	HDL-1	2.97(2.1-3.32)	3.02(2.425-3.6825)	2.74(2.31-3.59)	0.280
	HDL-2	1.24(0.85-1.52)	1.205(0.88-1.5925)	1.11(0.74-1.49)	0.232
	HDL-3	1.15(0.795-1.525)	1.105(0.765-1.505)	1.02(0.67-1.45)	0.607
	HDL-4	2.61(2.075-3.115)	2.645(1.96-3.375)	2.78(2.21-3.33)	0.765
Triglyceride	Total TG	118.27(91.665-149.355)	101(72.375-136.19)	105.57(85.7-154.22)	0.107
	VLDL	8.47(5.4-12.21)	6.095(2.3125-11.8425)	7.26(3.54-13.9)	0.086
	IDL	17.22(12.96-25.41)	17.89(14.5175-22.6525)	19.7(15.97-24.06)	0.231
	LDL	12.62(10.76-15.77)	11.35(8.6975-14.17)	11.31(9.44-14.19)	0.009 **
	HDL	79.74(58.885-100.06)	68.245(48.155-96.35)	71.06(58.34-109.19)	0.137
	VLDL-1	32.18(21.26-50.19)	28.255(19.6125-46.325)	33.5(20.58-51.36)	0.336
	VLDL-2	14.27(8.865-20.545)	11.63(7.2975-18.15)	12.38(9.07-19.92)	0.123
	VLDL-3	13.48(8.405-19.19)	10.9(7.29-15.645)	12.31(7.29-17.01)	0.204
	VLDL-4	10.73(7.075-12.98)	8.465(6.68-11.1525)	8.16(6.35-11.26)	0.081
	VLDL-5	2.95(2.505-3.665)	2.63(2.205-3.0925)	2.41(2-2.77)	<0.001 **
	LDL-1	5.63(4.17-7.94)	4.615(3.155-6.28)	5.05(3.61-6.67)	0.013 *
	LDL-2	2.2(1.74-2.86)	1.95(1.5275-2.4925)	2.07(1.56-2.63)	0.113
	LDL-3	1.47(0.95-2.025)	1.6(1.1975-2.135)	1.65(1.11-2)	0.455
	LDL-4	2.25(1.28-3.1)	2.5(1.73-3.2025)	2.59(1.94-3.1)	0.108
	LDL-5	2.2(1.38-3.295)	2.73(1.9875-3.775)	3.11(2.22-3.98)	0.009 **
	LDL-6	2.74(2.18-4.25)	3.575(2.8975-4.6425)	4.04(3.1-4.82)	0.004 **
	HDL-1	4.4(3.64-5.705)	3.87(2.83-5.2)	3.73(2.95-5.12)	0.006 **
	HDL-2	2.34(1.97-3.03)	2.095(1.6375-2.66)	2.03(1.68-2.46)	0.007 **
	HDL-3	2.46(2.03-3.085)	2.125(1.63-2.715)	2.15(1.76-2.63)	0.007 **

	HDL-4	3.63(3.205-4.28)	3.235(2.6975-3.9375)	3.41(2.96-3.94)	0.017 *
Phospholipid	VLDL	22.17(16.08-26.925)	19.57(13.955-25.2475)	19.58(15.84-26.65)	0.217
	IDL	5.38(3.39-7.92)	5.2(2.6825-7.19)	5.86(4.01-8.01)	0.236
	LDL	44.63(30.775-55.73)	49.785(40.8225-61.495)	52.34(45.56-62.36)	0.010 *
	HDL	60.57(52.18-68.3)	57.375(50.055-67.37)	53.69(47.35-63.44)	0.034 *
	VLDL-1	5.87(3.595-9.365)	4.985(3.2075-8.33)	5.96(4.06-9.09)	0.225
	VLDL-2	3.53(2.33-5.315)	3(2.06-4.7575)	3.58(2.66-5.62)	0.144
	VLDL-3	4.45(3.265-6.655)	3.9(2.4875-5.6025)	4.27(2.72-5.77)	0.220
	VLDL-4	5.21(3.655-6.185)	4.31(3.1875-5.4025)	4.28(3.37-5.4)	0.091
	VLDL-5	2.26(1.885-2.655)	1.87(1.4575-2.29)	1.8(1.61-2.12)	0.002 **
	LDL-1	10.78(8.095-14.285)	9.13(7.4-11.44)	9.22(8.46-12.27)	0.020 *
	LDL-2	5.2(4.235-7.265)	5.58(4.0475-7.13)	5.95(4.38-7.66)	0.802
	LDL-3	5.06(3.865-7.335)	6.25(4.9725-8.2225)	6.76(5.12-9)	0.017 *
	LDL-4	6.04(3.71-8.555)	7.265(5.0975-10.0325)	8.37(5.85-10.23)	0.008 **
	LDL-5	6.05(4.02-8.88)	8.67(6.5375-11.65)	9.47(7.72-12.77)	<0.001 **
	LDL-6	8.75(6.835-12.305)	11.56(9.305-14.7775)	13.53(10.16-15.97)	<0.001 **
	HDL-1	15.7(12.19-20.37)	14.595(11.6375-18.6375)	13.93(11.14-17.31)	0.084
	HDL-2	10.49(8.535-13.875)	9.685(7.9975-12.3775)	8.44(7.06-10.83)	0.002 **
	HDL-3	11.53(9.95-13.93)	10.875(8.9075-12.8275)	9.92(8.15-12.11)	0.028 *
	HDL-4	19.65(18.05-21.65)	19.715(17.205-22.7575)	20.32(17.15-23.02)	0.903

*$P<0.05$;

**$P<0.01$
